# Construction of Molecular Model and Adsorption of Collectors on Bulianta Coal

**DOI:** 10.3390/molecules25174030

**Published:** 2020-09-03

**Authors:** He Zhang, Peng Xi, Qiming Zhuo, Wenli Liu

**Affiliations:** 1 School of Mining Engineering, Heilongjiang University of Science and Technology, Harbin 150027, China; zhe9127@163.com; 2 Department of Environmental Engineering, North China Institute of Science and Technology, Beijing 101601, China; 3 School of Chemical and Environmental Engineering, China University of Mining and Technology (Beijing), Beijing 100083, China; lwl@cumtb.edu.cn

**Keywords:** flotation, low-rank coal, coal molecular model, collector adsorption, molecular dynamics

## Abstract

To study the effects of different oxygen functional groups on the quality of flotation clean low-rank coal, two kinds of collectors with different oxygen-containing functional groups, methyl laurate, and dodecanol, were selected and their flotation behaviors were investigated. The Bulianta coal was the typical sub-bituminous coal in China, and the coal molecular model of which was constructed based on proximate analysis, ultimate analysis, ^13^C-NMR, and XPS. The chemical structure model of the coal molecule was optimized, and the periodic boundary condition was added via the method of molecular dynamics methods. The different combined systems formed by collectors, water, and a model surface of Bulianta coal have been studied using molecular dynamics simulation. The simulation results of dodecanol and methyl laurate on the surface of Bulianta coal show that dodecanol molecules are not evenly adsorbed on the surface of coal, and have higher adsorption capacity near carboxyl and hydroxyl groups, but less adsorption capacity near carbonyl and ether bonds. Methyl laurate can completely cover the oxygen-containing functional groups on the coal surface. Compared with dodecanol, methyl laurate can effectively improve the hydrophobicity of the Bulianta coal surface, which is consistent with the results of the XPS test and the flotation test.

## 1. Introduction

Low rank coal, such as lignite and sub-bituminous coal, makes up more than 50% of the world’s proven coal deposits [1]. With the increase of the world’s energy demand, the utilization of low-rank coal has become an urgent need and received widely attention [2]. The upgrading of low-rank coal to obtain high-quality coal resources has been conducted by the froth flotation method. This is a surface-based method based on the differences in surface properties between the target minerals and gangue minerals. In the process of froth flotation, the target minerals are often selectively hydrophobic by adding agents called collectors at the liquid/solid interface. The bubbles are injected into the flotation cell, and the hydrophobic minerals in the upper part of the flotation cell are recovered under strong stirring conditions [3,4,5]. In the process of slime flotation, the hydrophobicity of the coal particle surface is one of the most important factors to determine the flotation effect. However, the surface of low-rank coal is abundant with oxygen-containing functional groups (hydroxyl, carboxyl, carbonyl, phenolic hydroxyl), which will form a hydrogen bond with water molecules and lead to the low hydrophobicity of the coal surface [6]. The low hydrophobicity of low-rank coal limits the adsorption of conventional aliphatic hydrocarbon oil on the surface. It was proved that the introduction of oxygen-containing functional groups into oily collectors could make the collectors stably adsorb on the surface of low-rank coal, thus, changing the surface hydrophobicity of low-rank coal [7,8]. Zhou et al. [9] used Sorbitan Monolete (Span 80) series surfactants with hydroxyl and ester groups to explore the flotation effect of low-rank coal and found that it can significantly improve the hydrophobicity of low-rank coal. Jena et al. [10] found that alcohol collectors can effectively improve the hydrophobicity of low-rank coal. Xia et al. [11,12] investigated that fatty acid methyl esters in biodiesel have a better flotation effect than conventional hydrocarbon oil collectors. Thus, it is valuable to study the adsorption behaviors of the two collectors on low-rank coal and their effects on improving the flotation efficiencies of coal slime to find whether hydroxyl or ester groups is advantageous for reducing the hydrophilicity of low-rank coal.

Molecular dynamics simulation is based on the classical mechanical method to simulate the dynamics and thermodynamics properties of each molecule in the system. At present, the molecular dynamics method has become an important tool to provide the micro and basic understanding of the molecular system, which has been widely used in the field of mineral flotation reagents [13,14]. Wang et al. [15] described the adsorption behavior of dodecyl amine, sodium oleate, and their mixtures on the surface of Muscovite by molecular dynamics simulation. Rai et al. [16] used molecular dynamics simulation to describe the adsorption process of oleic acid and dodecyl amine hydrochloride on complex aluminosilicate minerals, such as spodumene, jadeite, feldspar, and mica.

All these researches reveal the reactivity and selectivity of collectors and their adsorption behavior on the mineral surface, which is of great significance to further understand the adsorption mechanism of collectors on the mineral surface. However, all these studies were mainly concentrated on minerals with a single structure and chemical composition. Coal is a kind of substance with complex structures and various chemical components. Due to its complex surface properties, the application of molecular simulation in the field of coal flotation is still in its infancy [17]. Zhang et al. [18] selected a wiser model to represent bituminous coal in order to simulate the adsorption behavior of three different types of the collector on the coal surface through MD, which provides a basis for the study of the adsorption behavior of collector on the coal surface. Lyu et al. [19] combined the experiment and molecular dynamics simulation method to study the absorption behavior of NPEO-12 on the surface of Hatcher sub-bituminous coal.

However, due to the limitations of previous coal models (such as Wiser and Hatcher models) in describing the heterogeneous structure of coal, their surface properties may be different from the coal used in the test. The difference between coal surface properties will lead to the difference in collector adsorption behavior. It is necessary to construct a reasonable molecular structure of coal to explore the interaction between coal and collector.

To conduct a comparative study with the experiment, researchers began to construct the coal structure model based on its surface properties used in the experiment. Moreover, it is widely used to explore the adsorption behavior of coalbed methane on the coal surface. Lin et al. [20] used solid-state ^13^C nuclear magnetic resonance (NMR), Fourier transform infrared spectra (FTIR), and X-ray photoelectron spectroscopy (XPS) to analyze the surface properties of Indonesian lignite, and constructed the macromolecular structure model of Indonesian lignite. The calculated chemical displacement spectrum of the model is in good agreement with the experimental results. Meng et al. [21] used a proximate analysis, ultimate analysis, XPS, ^13^C-NMR to construct the Zhaozhuang coal molecular model. The GCMC method was used to study the adsorption behavior of methane in coal molecules. The relative adsorption error between simulation results and test results is only 3.303%.

In the field of coal flotation, few studies on the adsorption behavior of collectors on the coal surface by constructing the coal model based on the surface properties of the coal used in the experiment. Accurately constructing the molecular structure of coal is the basis of exploring the interaction between coal and collector, which also helps to determine the interaction between different oxygen-containing functional groups (hydroxyl and ester groups) and the coal surface, and then to select an effective collector suitable for sub-bituminous coal.

The purpose of this study is to screen out the suitable non-ionic collector for reducing the strong hydrophilicity of sub-bituminous coal from Bulianta mine by molecular dynamics method and to study the influence on the wettability of Bulianta coal. In this paper, the surface properties of the Bulianta coal were explored, and the corresponding coal structure model was established. A molecular dynamics simulation was adopted to investigate the micro-adsorption behavior of dodecanol and methyl laurate, where the number of nonpolar groups is the same, but with different polar groups. The results of experiments and simulations were compared.

## 2. Results and Discussion

### 2.1. Coal Molecule Construction Results

#### 2.1.1. Conventional Analysis

The results of conventional analysis from the Bulianta coal sample were shown in Table 1.

The atomic number ratio from the Bulianta coal sample was shown in Table 2.

#### 2.1.2. Solid ^13^C Nuclear Magnetic Spectrum Analysis

According to the chemical shift attribution of the ^13^C-NMR spectrum, the obtained spectrum was fitted with peak fitting by the Origin software, and the 12 structural parameters of Bulianta coal were obtained [22,23], as shown in Table 3.

The ratio of bridged aromatic carbon ($f_{a}^{B}$) to surrounding carbon ($f_{a}^{H}+f_{a}^{P}+f_{a}^{S}$) was defined as $X_{BP}$, which had important reference value for determining the type and quantity of aromatic rings in coal [20]. The $X_{BP}$ value of Bulianta coal was 0.26.

The value of $X_{BP}$ for several simple aromatic rings, such as 2, 3 cyclic aromatic hydrocarbons, was 0.25 and 0.4, respectively. The value of $X_{BP}$ for the Bulianta coal was between that for 2 ring aromatic hydrocarbons and 3 ring aromatic hydrocarbons. Since the 1970s, it has been found that the aromatic rings of the basic structural units of low coal rank coal are mainly benzene ring, naphthalene ring, and phenanthrene ring [24]. From this, it can be estimated that the main aromatic structure of Bulianta coal is 1–3 ring. Considering that ACD/CNMR prediction software can only calculate the molecular number of 256 atoms (excluding the number of H atoms), constantly adjust the number and type of aromatic structure in the Bulianta coal model, so that the $X_{BP}$ of the model is consistent with the $X_{BP}$ obtained in the experiment, and finally determine the aromatic structure and type in the coal sample, as shown in Table 4. Thus, the number of aromatic carbons in the coal of Bulianta is 87.

According to the results of ^13^C solid-state NMR, $f_{a}$ is 62.24, so the total number of carbon atoms in the structure model is 143, and the number of fatty carbon and carbonyl (carboxy) carbon is 56. It could be seen from Table 3 that the methylene and methine carbon rate ($f_{al}^{H}$) of Bulianta coal is 37.76. Methylene is the main structure to make up the aliphatic structures chain and alicyclic rings. While the methyl carbon rate ($f_{al}^{*}$) is only 6.90, which is means that the aliphatic carbon in the Bulianta mainly existed in the forms of alicyclic rings and long aliphatic chains. The Atomic number ratio of H/C in Table 2 is 0.84. In the subsequent modeling process, the type of carbon atom should also be adjusted according to the H/C ratio, so that it corresponds to the H/C ratio of the experimental value.

#### 2.1.3. XPS Analysis of Bulianta Coal

**Figure 1:**
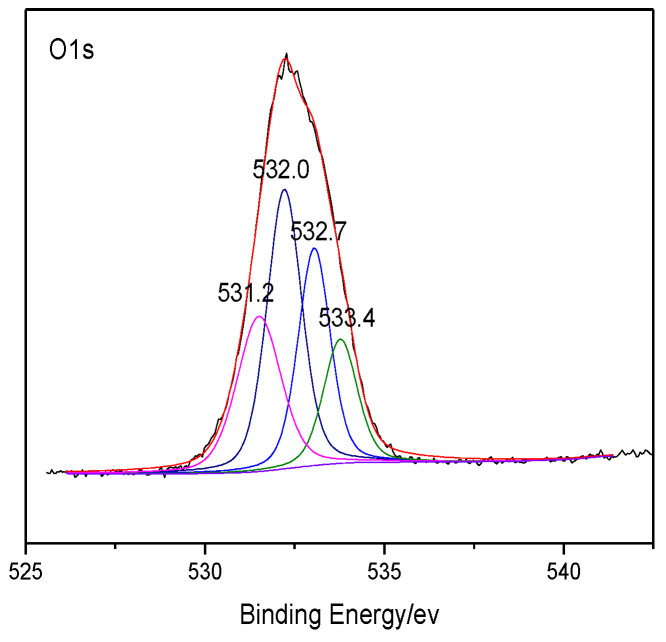
XPS O 1s spectrum of Bulianta coal.

**Figure 2:**
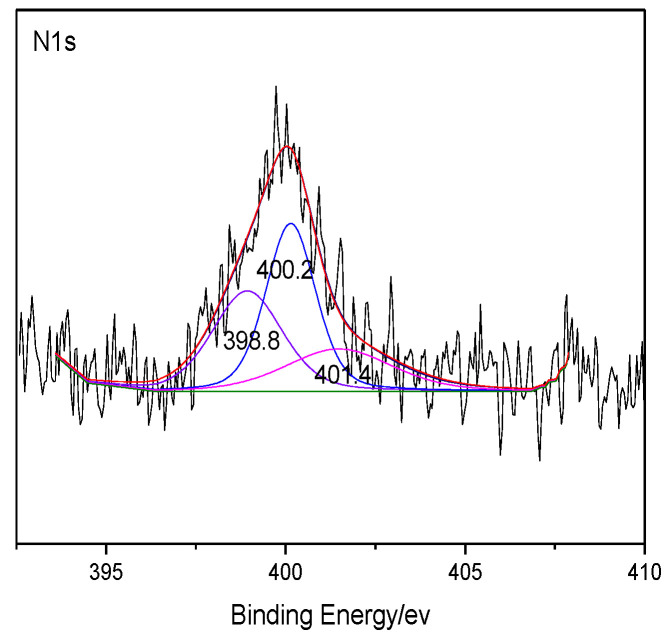
XPS N 1s spectrum of Bulianta coal.

The heteroatoms within coal were analyzed through XPS, which can make quantitative analysis for elements like O and N on the coal surface without destructing the sample structure [25,26,27]. The XPS results of coal are shown in Figure 1 and Figure 2. The peaks of several elements are processed by Origin software, and the distribution of the corresponding heteroatom functional groups are shown in Table 5. According to the distribution law of oxygen-containing functional groups, the results of ultimate analysis, and the number of carbon atoms previously determined, it can be determined that the number of O atoms and N atoms in the model of Bulianta coal is 29 and 2, respectively. Combining the results of XPS, there are 10 oxygen atoms presented in the form of hydroxyl groups (-OH), six oxygen atoms presented in the form of ether bonds (-O-), five oxygen atoms presented in the form of carbonyl groups (C=O), and four oxygen atoms presented in the form of carboxyl groups (COO-) in the model of Bulianta coal. The two nitrogen atoms in the coal model of Bulianta coal exist in the form of pyridine and pyrrole, respectively.

#### 2.1.4. Construction of Plane Structure Model of Coal

**Figure 3:**
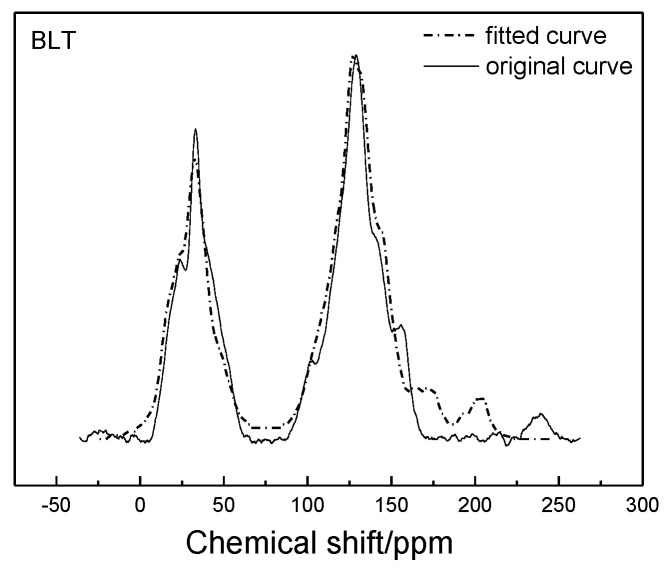
Experimental and calculated ^13^C-NMR spectrum of the Bulianta coal.

**Figure 4:**
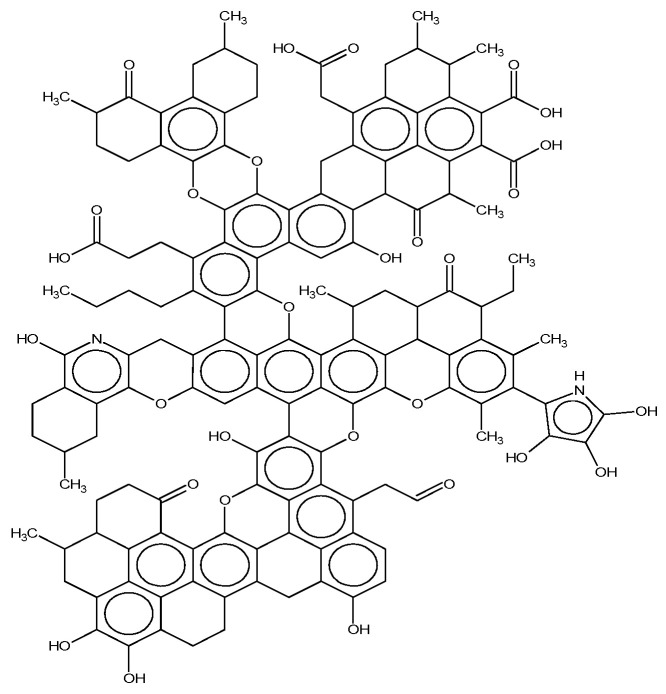
The plane structure model of the Bulianta coal.

Based on the structure of aromatic carbon, aliphatic carbon, and heteroatom determined by ^13^C-NMR and XPS, the initial model of the Bulianta coal samples was constructed by combining the structural parameters of ultimate analysis and the ratio of H/C and O/C atoms. There are many isomers in coal, due to the different connection ways, so it is necessary to adjust the connection ways of carbon in coal. The ACD/CNMR Predictor software was used to calculate the ^13^C-NMR spectrum of the structure model. The molecular structure of the coal was constantly revised by comparing the differences between the calculated spectrum and experimental spectrum, so that the calculated spectrum is in conformity with the experimental spectrum, as shown in Figure 3 [28]. Finally, the plane structure models of Bulianta coal were determined, as shown in Figure 4 and Table 6.

### 2.2. Construction of 3D Structure Model of Coal

**Figure 5:**
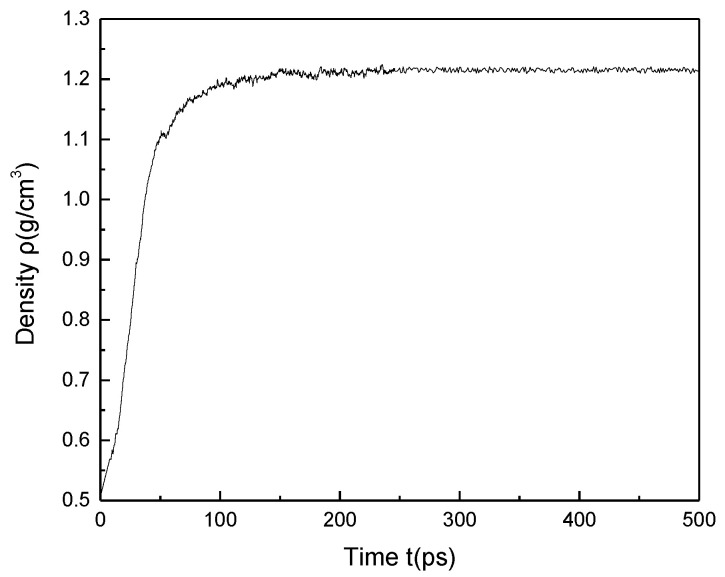
Change of calculated density with time.

**Figure 6:**
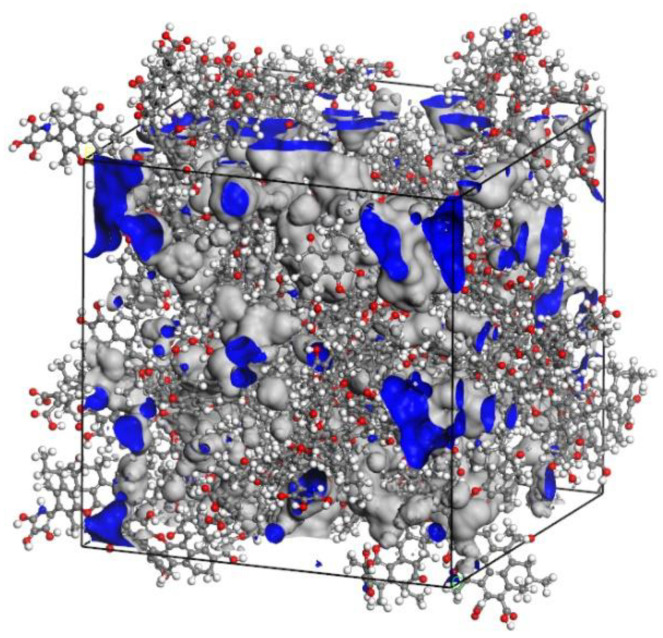
The 3D structure model of Bulianta coal (45 Å × 45 Å × 45 Å).

Through the methods described above, the 20 minimum energy plane structure models of coal were randomly placed into the cubic structure box to obtain a low-density 3D structure model. Then 500 ps molecular dynamics simulations were performed to adjust the crystal cell structure continuously using the NVT ensemble and NPT ensemble, so that the density of coal molecular structure reached a balance, as shown in Figure 5. The final 3D structure model of Bulianta coal was also obtained, as shown in Figure 6. Therefore, the 3D structure model of the coal with a density of 1.22 g/cm^3^ is obtained. The above density is the molecular weight of coal divided by the volume of the crystal cell, but there are many pores (blue part in Figure 6) of different sizes on the surface of coal. Actually, in the measurement of the coal density, helium is often used as the detection gas to remove the pores in the coal to get the density of coal. The measured density of the Bulianta coal is 1.39 g/cm^3^. Therefore, the simulated helium density of Bulianta coal was calculated by using Atom Volumes and Surfaces in Material Studios software, and helium gas is used as the detecting gas with a dynamic radius of 1.29 Å [29]. The simulated helium density of the 3D structure model is 1.35 g/cm^3^, which is close to the density of 1.39 g/cm^3^ measured in the experiment. So the molecular configuration of Bulianta coal is considered to be relatively reasonable, which can be used for further study of collector adsorption behavior.

## 3. Molecular Dynamics Simulations

### 3.1. Selection of Collector

**Figure 7:**
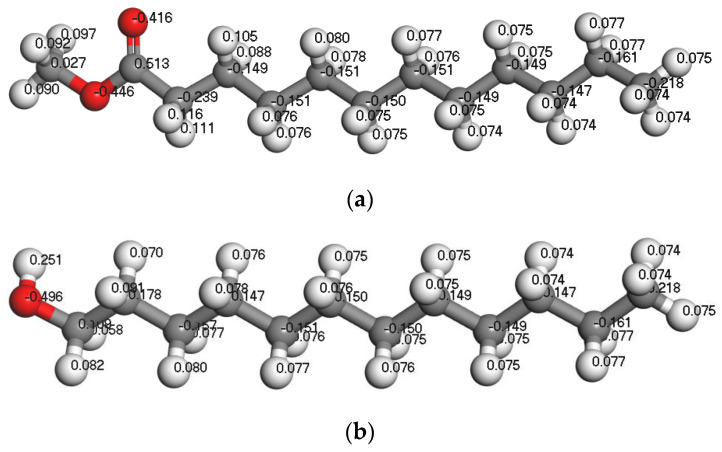
The structures of the selected collectors: (**a**) methyl laurate and (**b**) dodecanol. The representation of gray, white, and red is the C, H, and O atom, respectively.

The difference in collector performance is mainly due to its different structures. Dodecanol and methyl laurate were selected to compare the effect of ester and alcohol collectors on the wettability of the coal surface after the adsorption. The Mulliken charge distribution of two collectors is shown in Figure 7. It can be seen from Figure 7 that the charge of the oxygen atom in the two collectors is almost the same (−0.416 ~ −0.496e). The charge of H linked to the oxygen in the hydroxyl group of dodecanol is 0.251, which is much higher than that in the ester of methyl laurate. It means that dodecanol has a stronger electrostatic interaction with water than methyl laurate. Dodecanol will form a hydrogen bond with an oxygen atom in water and shows strong hydrophilicity.

### 3.2. Adsorption Configuration of Collectors on the Surface of Bulitana Coal

**Figure 8:**
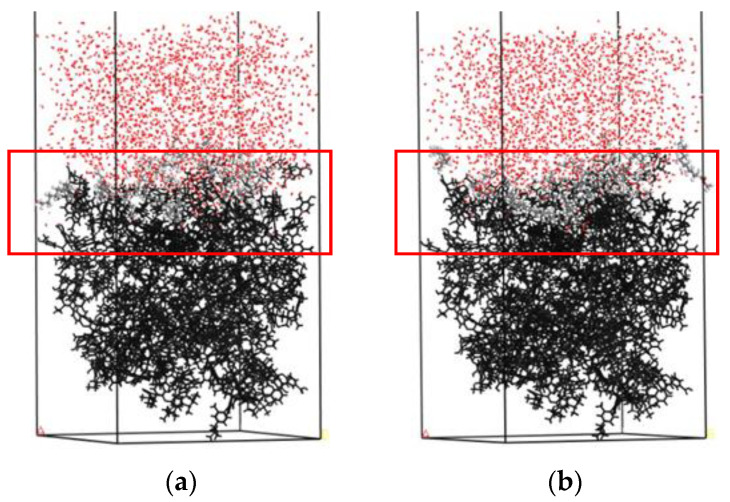
The equilibrium adsorption configuration on the surface of Bulianta coal in an aqueous environment: (**a**) Water/methyl laurate/coal system and (**b**) water/dodecanol/coal. For clarity, the coal surface models are shown as black.

**Figure 9:**
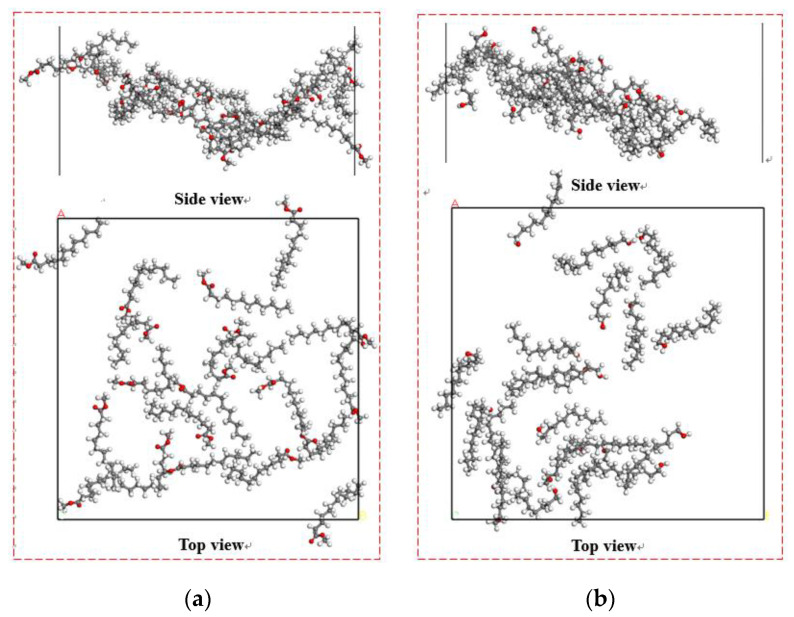
The spatial equilibrium structures of collectors adsorbed on Bulianta coal. (**a**) Methyl laurate; (**b**) dodecanol.

Figure 8 shows the equilibrium adsorption configuration of the collector molecular layer on the coal surface in the liquid phase. Through the analysis of the collector structure at the coal water interface, the molecular morphology of the collector can be obtained directly. In order to observe the spatial equilibrium structures of collectors adsorbed on the coal surface at equilibrium, the reagent layer (red box) in Figure 8 is enlarged, as shown in Figure 9.

It can be seen from Figure 9 that different collectors have a different degree of aggregation on the coal surface, and the dodecanol molecules with strong hydrophilicity have stronger aggregation degrees than methyl laurate.

### 3.3. The Density Distribution of Molecules at Different Distances along Z axis

**Figure 10:**
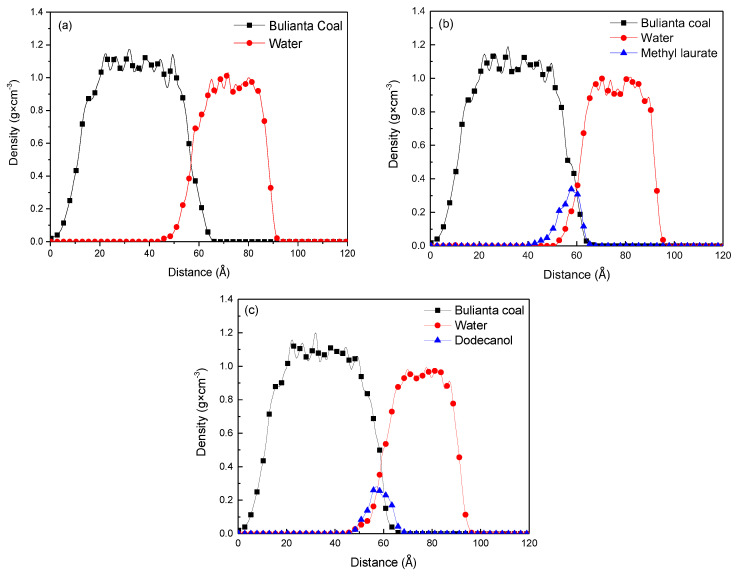
Mass density distributions of coal, collectors, and water: (**a**) Water/coal, (**b**) water/methyl laurate/coal, and (**c**) water/dodecanol/coal system.

The density profile of each molecule at different distances along *Z*-axis was obtained by molecular dynamics simulation to determine the spatial position and directional distribution of water, collector, and coal molecules in each system. The Z axis is perpendicular to the coal surface, and the direction is vertical upward. The density distribution at different distances along the *Z*-axis is divided into many equal intervals along the normal direction of the surface. The total atomic mass and volume of each interval are taken to obtain the density of atoms in the interval at a distance. The density of all regions is counted, as shown in Figure 10.

**Figure 11:**
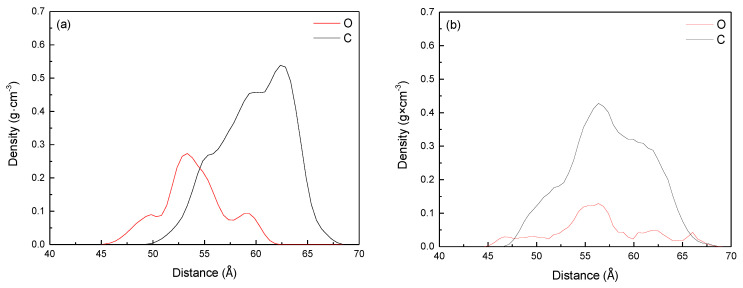
Density profiles of hydrophobic tails and hydrophilic head groups of collectors: (**a**) Methyl laurate; (**b**) dodecanol.

It can be seen from Figure 10 and Table 7 that the density distribution of Bulianta coal molecules is not affected by the adsorption process, and there is consistent density distribution in several systems. From the density distribution range of collectors, it can be seen that methyl laurate has a narrow density distribution in *Z*-axis compared with dodecanol. It means that methyl laurate has a wide distribution on the X × Y plane perpendicular to the Z axis (parallel to the coal surface), when the two collectors have the same amount. It also can be confirmed from the distribution range of water molecules in *Z*-axis. Under the action of dodecanol molecules, the density distribution of water molecules is closer to coal molecules, which means that dodecanol molecules are difficult to effectively cover coal surface compared with methyl laurate. In order to determine the molecular orientation of the collector at the coal water interface, the density distribution of the polar head group and hydrophobic tail group along the Z axis in the equilibrium state is determined by simulation method, as shown in Figure 11.

It can be seen from Figure 11 that the mass density distribution range (44.49–63.28 Å) of the polar head group (ester group) is closer to the coal surface than that of the hydrophobic tail group (fatty chain) (49.24–67.85 Å). It means that the polar head group deflects the coal surface during the molecular adsorption of methyl laurate. The fatty chain is far away from the coal surface and exposed to the water phase, making the coal surface more hydrophobic. However, the mass density distribution range of the polar head group of dodecanol molecular adsorption is 44.58–69.48 Å, which is larger than that of the fatty chain (46.88–68.69 Å). The density curve of the polar head group of dodecanol is closer to the coal surface as a whole. This means that when the dodecanol molecules are adsorbed on the coal surface, most of the polar head groups of dodecanol turn to the coal surface, while a few of the polar head groups turn to the water phase.

### 3.4. The Radial Distribution Functions

**Figure 12:**
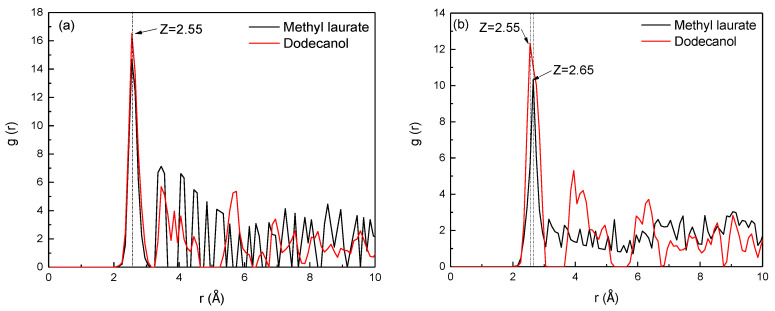

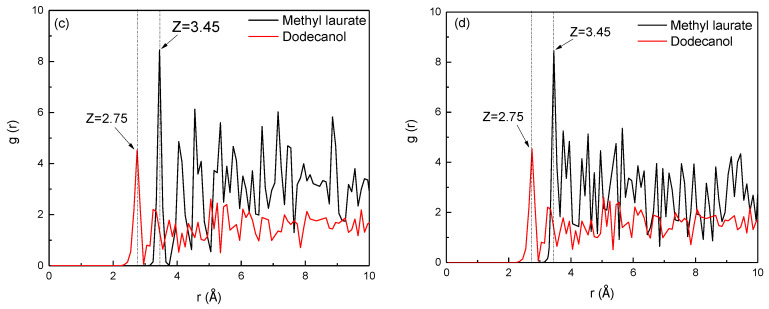
RDFs between the collector oxygen atoms and functional groups of Bulianta coal. (**a**) Carboxyl; (**b**) hydroxyl; (**c**) carbonyl; (**d**) ether bond.

The radial distribution function (RDF) between atoms can determine the aggregation degree of collectors on different functional groups of Bulianta coal, and then determine the adsorption law of collectors on different sites of Bulianta coal. The position and intensity of the first peak formed by the interatomic radial distribution function represent the relative position and order degree between the collector molecule and the coal surface functional group, respectively [30]. Figure 12 shows the RDF between the oxygen atom in the polar groups of collectors and the oxygen atom of the oxygen functional group on the surface of Bulianta coal.

Figure 12a shows the radial distribution function between the oxygen atom in the two collectors and the oxygen atom of the carboxyl group on the surface of Bulianta coal. It can be seen from Figure 12a that the peak positions of the two collectors and the carboxyl groups on the coal surface appear at 2.55 Å, but the peak value of dodecanol here is higher than methyl laurate. It means that dodecanol has a higher degree of aggregation on the carboxyl group surface than that of methyl laurate.

Figure 12b shows the radial distribution function between the oxygen atoms of the two collectors and the oxygen atoms of hydroxyl on the surface of the Bulianta coal, which can reflect the aggregation degree of the two collectors near the surface hydroxyl of the Bulianta coal. It can be seen from Figure 12b that the RDF peak formed by dodecanol oxygen atoms and coal surface hydroxyl group appears at 2.55 Å, while methyl laurate appears at 2.65 Å, which may be due to the stronger interaction between dodecanol and coal surface hydroxyl group. At the same time, the RDF peak formed by dodecanol and coal surface has a higher peak, which means that dodecanol has a higher degree of aggregation than methyl laurate near the hydroxyl of the coal surface.

The RDF peak of dodecanol near the carbonyl and ether bonds on the coal surface appears at 2.75 Å, while the RDF peak of methyl laurate near the carbonyl and ether bonds appears at 3.45 Å (Figure 12c,d). This may be due to the strong hydrogen bond between the highly polar dodecanol and the carbonyl or ether bonds on the coal surface, while the weak hydrogen bond between methyl laurate and the carbonyl or ether bonds on the coal surface. However, the peak strength of methyl laurate is much higher than that of dodecanol, which may be due to a part of the polar head base of dodecanol deflecting towards the water phase, resulting in the decrease of dodecanol adsorbed on the coal surface.

In conclusion, the adsorption of dodecanol molecules with strong polarity on the coal surface is unbalanced. The hydrogen bond between dodecanol and carboxyl or hydroxyl groups on the coal surface is strong, but the hydrogen bonding between dodecanol and carbonyl or ether bond is relatively weak. Most of the dodecanol molecules preferentially adsorb near the carboxyl or hydroxyl groups, and the adsorption capacity near the carbonyl or ether bonds is small. Dodecanol molecules have higher adsorption capacity near carboxyl and hydroxyl groups, but less adsorption capacity near carbonyl and ether bonds. However, the hydrogen bonding between methyl laurate and various oxygen-containing functional groups on the coal surface is similar, which leads to more uniform adsorption of methyl laurate on the coal surface.

### 3.5. Mobility of Water Molecules before and after the Adsorption of Collectors

The different adsorption behavior of collectors on the surface of Bulianta coal will affect the diffusion behavior of water molecules on the surface of coal. This diffusion behavior can be reflected by the mean square displacement (MSD) and diffusion coefficient (d) of water molecules on (modified) coal surface [31,32,33].

**Figure 13:**
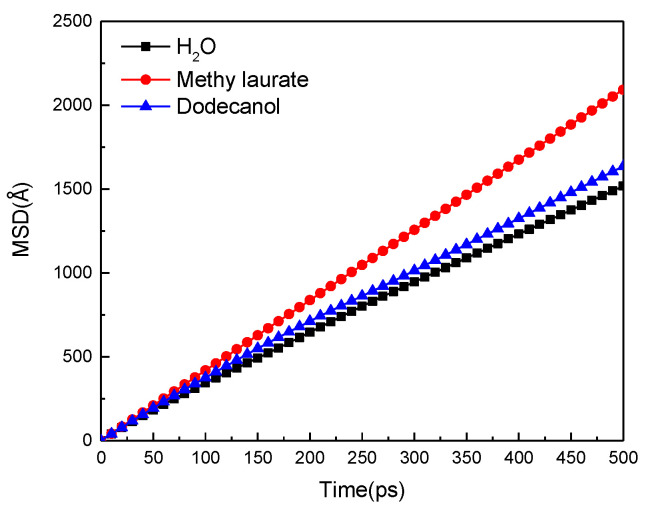
Mean square displacement (MSD) curves of water molecules.

MSD can quantify the diffusion strength of water molecules on the coal surface over time, obtained by molecular dynamics simulation, as shown in Figure 13. The diffusion strength of water molecules on the (modified) coal surface gradually increases with the extension of simulation time, and the enhancement range follows that of methyl laurate > dodecanol.

In order to further determine the diffusion degree of water molecules adsorbed on (modified) coal surface, the diffusion coefficient was calculated by Einstein’s equation.

(1) $$D=\frac{1}{6}\underset{t\to \infty }{\lim}\frac{d}{dt}\left(MSD\right)=\frac{1}{6}K_{MSD}$$

Here, *K_MSD_* is the slope of the *MSD* curve.

According to Equation (1), the self-diffusion coefficient (D) of water in water/coal and water/collector/coal systems can be determined. Table 8 shows the diffusion coefficient of water molecules after the collector adsorbs on the surface of Bulianta coal.

According to Table 8, the self-diffusion coefficient of water molecules on the surface of raw coal is 4.98 × 10^−9^ m^2^/s. The self-diffusion coefficient of water molecules increases when the surface of Buianta coal is covered by two kinds of collector molecules. It shows that many polar groups on the surface of Bulitana coal have a strong hydrogen bond effect on the polar water molecules, which limits the movement of water molecules. When the collector is adsorbed on the surface of Bulianta coal, the collector covers the polar groups on the surface of coal, which weakens the restriction of the coal surface on water molecules and improves the mobility of water molecules on the surface of the modified coal. The high mobility of water molecules is conducive to the removal of water molecules on the coal surface by bubbles, which facilitates the attachment of bubbles on the coal surface. Therefore, the enhancement range of the hydrophobicity of the two collectors to the surface of Bulianta coal is methyl laurate > dodecanol.

### 3.6. XPS Analysis of Collector before and after Adsorption

**Figure 14:**
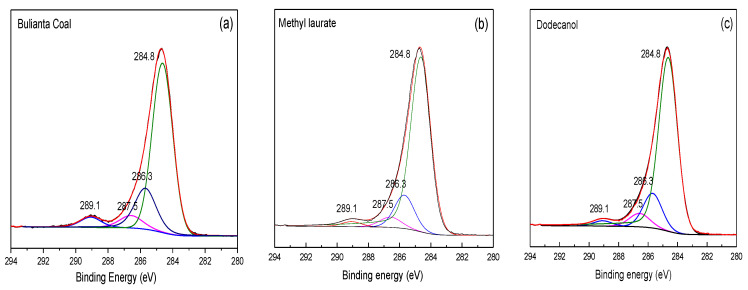
XPS C 1s spectrum of (**a**) raw coal, (**b**) the coal surface after the adsorption of methyl laurate, (**c**) the coal surface after the adsorption of dodecanol.

As important elements widely existing on the coal surface, the relative content of carbon and oxygen directly reflects the number of oxygen functional groups on the coal surface. The carbon spectrum could determine the relative content of C-C or C-H and oxygen-containing functional group in coal. Therefore, it is necessary to study the changes of carbon and oxygen on the coal surface before and after chemical adsorption for obtaining the covering effect of different collectors on oxygen functional groups on the coal surface. Figure 14 shows the XPS C 1s spectra before and after the adsorption of collectors. The relative content of carbon on coal surface before and after collector adsorption is recorded in Table 9.

It can be seen from Table 9 that under the action of two collectors, the carbon content in coal increases, while the oxygen functional group content decreases relatively, which shows that both collectors can effectively reduce the number of oxygen functional groups on the surface of Buianta coal. The content of C-O in Bulianta coal decreased from 19.8% to 16.56% after dodecanol adsorption, which was higher than that of C-O on the surface of coal after methyl laurate adsorption. On the one hand, it is difficult for dodecanol to cover the ether bond. On the other hand, it is possible that dodecanol has a strong covering effect on the coal surface hydroxyl, but a small part of the hydroxyl deviates from the coal surface, which makes the reduction of coal surface hydroxyl unclear.

After the adsorption of dodecanol, the change of the carbonyl content on the coal surface is not obvious, while the carboxyl group is greatly reduced. According to the simulation, it can be seen that this is due to the unbalanced adsorption of dodecanol. Dodecanol is mainly adsorbed near the hydroxyl and carboxyl groups on the coal surface, while the adsorption amount is small near the carbonyl group on the coal surface. As a result, the change of the carbonyl group is not obvious before and after the adsorption of dodecanol, while the carboxyl group is greatly reduced. The oxygen-containing functional groups on the coal surface decreased to a certain extent after the adsorption of methyl laurate, which was due to the relatively balanced adsorption of methyl laurate on the coal surface, which was consistent with the simulation results.

### 3.7. Flotation Test Results

**Figure 15:**
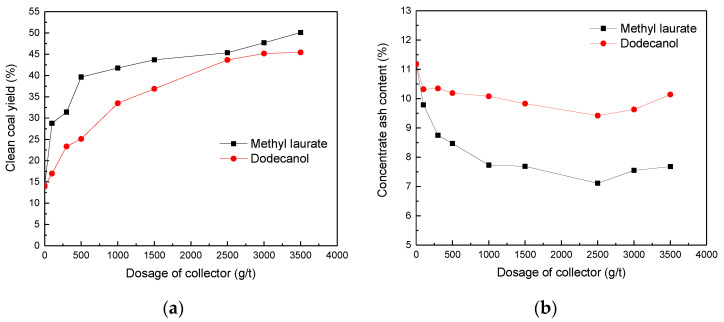
Effect of two kinds of collectors on Bulianta coal flotation. (**a**) Clean coal yield, (**b**) concentrate ash content.

Figure 15 shows the effects of two kinds of collector contents on Bulianta coal flotation. With the increase of collector content, clean coal production began to increase significantly. Without the addition of collector, the yield of Bulianta clean coal is 14.01%, and the ash content is 11.18%. The ash content first decreases with the increase of collector content. This result may be due to the existence of a certain degree of sliming phenomenon in raw coal. After the coal is immersed in water, the ash is mixed into the clean coal. With the increase of collector content, the proportion of combustible carbon in clean coal products gradually increases, and the proportion of ash gradually decreases. When the amount of collector reaches 2500 g/t, the ash content continues to increase with the increase of the amount of collector. Comparing the flotation results of two collectors, it can be found that the clean coal yield of methyl laurate is much higher than that of dodecanol. Accordingly, the concentrate ash content of methyl laurate are much lower than that of dodecanol. Those above can safely conclude that methyl laurate may be more suitable for low-rank coal flotation.

## 4. Experiment and Simulation Methods

### 4.1. Coal Molecular Model

#### 4.1.1. Sample and Preparation

The typical sub-bituminous coal was selected, which was obtained from the Bulianta coal mine (Erdos, Inner Mongolia, China). Some coal samples were deashing by using of the float-sink method. The sample with a density of 1.3–1.4 kg/m^3^ was selected. The sample with the density of 1.3–1.4 kg/m^3^ has less ash, which will not affect the accuracy of the instrument. The acquired samples were analyzed using proximate analysis, XPS experiment, and solid-state ^13^C-NMR. Demineralization of the other coal sample was performed using HCL and HF to minimize the mineral effect during the analysis. Next, a large amount of deionized water to rinse the sample until it becomes neutral and dry the sample to a constant weight [34,35]. This part of the sample could be used for the true density test for coal, which requires less ash content in the sample.

The dodecanol (purity: 99%; formula: C_12_H_26_O) and methyl laurate (purity: 99%; formula: C_13_H_26_O_2_) were obtained from Shanghai Macklin Biochemical Co., Ltd., Shanghai, China.

#### 4.1.2. Conventional Analysis

The proximate analysis of the raw coal sample was conducted according to the method of GB/T212-2008. The ultimate analysis for the demineralized sample was determined using Elementa’s Vario EL cube CHNOS elemental analyzer. The content of carbon, hydrogen, nitrogen, and sulfur is the average value of two parallel samples, and the content of oxygen was calculated by the difference value method. The true density of coal was obtained by AccuPyc 1330 fully automatic density analyzer, and was determined by gas (helium) displacement technology.

#### 4.1.3. Solid-State ^13^C-NMR Spectroscopy

The solid-state ^13^C NMR spectroscopy was obtained from a Bruker AVANCE III HD 500 MHz spectrometer, operating at the resonant frequency of ^13^C nucleus of 100 MHz using a double-resonance solid-state NMR probe and a 4 mm diameter ZrO_2_ rotor. A cross-polarization (CP) magic-angle spinning experiments was employed using 0.05 s sampling time, 4 s pulse width, MAS speed of 8 kHz, 5 s cycle delay time, and 7000 scans. Then the cross-polarization (CP) technique with total side band suppression (TOSS) was conducted.

#### 4.1.4. X-ray Photoelectron Spectroscopy (XPS)

The XPS analysis was conducted on Thermo Scientific Escalab 250 Xi X-ray photoelectron spectrometer. The AlK Alpha anode was used, the power was 200 W. The transmittance of wide spectrum scanning was 100 eV, and the step length was 1000 meV; the transmission energy of fine spectral scanning was 30 eV, and the step length was 50 meV. Before data analysis, 284.8 eV was selected as the C 1s spectral peak calibration standard for calibration [26,36].

### 4.2. Simulation Details

#### 4.2.1. Optimizing the Coal Molecular Structure

The plane structure model of coal was constructed based on proximate analysis, ultimate analysis, ^13^C-NMR, XPS, and then it was optimized by the Molecular mechanic calculations in the Forcite module of Material Studio 2018 software. Molecular mechanics used the method of Smart Minimizer. The convergence standard was fine, and the charge balance method (QEq) was used to obtain the atomic charge. The calculation of electrostatic force and van der Waals force adopted the atomic state. In order to overcome the energy barrier of the molecular structure and make the energy of the system reach the optimal geometric state, anneal kinetics calculation was carried out, and the temperature control program selects the Nose [37]. The initial temperature was set to 298 K and then rose every 60 K until the temperature reached 1098 K. After the temperature reaches 1098 K, the temperature is cooled at the rate of 60 K/time until the temperature drops to 298 K. The final temperature returns to 298 K, which is the experimental temperature. The number of annealing cycles is ten.

The optimized plane structure model of coal was randomly put into the cube structure box. A low-density 3D structure model with a density of 0.5 g/cm^3^ was constructed to prevent the overlap of polycyclic aromatic hydrocarbons and functional groups in coal. To achieve an appropriate density of Bulianta coal, geometric optimization of the initially constructed units was carried out, including: (1) Annealing recycle from 298 to 1098 K using NVT ensemble; (2) NPT ensemble was used to compress and decompress at 0.01 Gpa and 0.1 Mpa, respectively [29]; (3) optimization of coal model structure by energy minimization. At last, the 3D structure model of the coal was obtained. All of the molecular dynamic simulations uses the Nose temperature control method [37]. The NPT pressure control uses the Berendsen method. The simulation time was 500 ps, and the time step was 1.0fs. Ewald method was used for the electrostatic force with an accuracy of 1 × 10^−4^. An atom-based method was used for van der Waals force with a cut-off distance of 15.5 Å.

#### 4.2.2. MD Simulations for Coal/Collector/Water System

The Bulianta coal model was packed into a 3D cell of 45Å × 45Å (X × Y). The water molecules were simulated using the SPC (simple point charge) model. The single water, dodecanol, and methyl laurate molecules were geometrically optimized using Dmol3. The generalized gradient approximation (GGA) functional is chosen, the electron exchange-correlation potential is PW91 functional, and double numerical plus polarization (DNP) basis set is selected for all atoms. The electron spin is not limited during the simulation. The DIIS method is used to accelerate the SCF convergence. The smealing standard used in orbit calculation is 0.005hartree. The accuracy of geometric optimization is fine.

The collector cell containing 10 collector molecules and water slab containing 1000 water molecules were constructed with the same length and width to the coal surface in the same way. A vacuum layer with a thickness of about 110 Å was added to the top of the coal/collectors/water system to avoid the interaction between the top surface and the bottom surface of the model caused by periodic boundary conditions. The MD simulation with a simulation time of 10 ns and a time step of 1 fs was carried out by the system at 298K. The force field used in the calculation is the COMPASS II (Condensed-phase Optimized Molecular Potentials for Atomistic Simulation Studies II) force field, which extends the coverage of polymer and drug-like compounds in the material force field based on the COMPASS force field [38]. Finally, 500 ps simulations were conducted to calculate the final results after the system is balanced.

### 4.3. Flotation Procedure

The flotation tests were conducted in a cell of 1 L with a pulp concentration of 80 g/L at 25 °C. For each test, the dosage of 2-octanol frother was 200 g/t. At first, the coal sample was added into the cell filled with 1 L of deionized water. Then, the collector was added into the flotation pulp, and the pulp was carried out under stirring at an impeller speed of 2000 rpm for 2 min. At last, 2-octanol frother was injected for contact times of 30 s. The airflow rate was 0.2 m^3^/h, and the concentrate product was collected for 3 min. The concentrate and tailings were filtered in an oven at 80 °C and dried to constant weight, and then ash content was measured.

## 5. Conclusions

In order to select the collector suitable for low-rank Bulianta coal, both methyl laurate and dodecanol (with the same carbon number and different polar groups) are selected in this paper. According to the relevant structural parameters obtained from the experiment, Bulianta coal molecular model is established, and the structure is optimized. The 3D structure model is constructed by molecular dynamics, and the density calculation is carried out, which is in good agreement with the actual density.

The different adsorption behaviors of the two collectors on the surface of Bulitana coal were determined by molecular dynamics. The results show that dodecanol had a higher adsorption capacity near the carboxyl and hydroxyl groups, but less near the carbonyl and ether bonds. Some of the polar groups of dodecanol deviate from the coal surface and tend to the water phase. However, the adsorption of methyl laurate on several oxygen-containing functional groups on the coal surface is relatively uniform. The polar groups of methyl laurate are all toward the coal surface. The methyl laurate containing ester group with weak hydrophilicity can more effectively improve the surface hydrophobicity of Bulianta coal compared with dodecanol containing hydroxyl with strong hydrophilicity.

XPS and flotation results show that methyl laurate can effectively reduce the amount of oxygen-containing functional groups in coal compared with dodecanol. Dodecanol is mainly adsorbed near the carboxyl and hydroxyl groups on the coal surface, but less on the carbonyl group. The adsorption of methyl laurate on the coal surface is more uniform, which makes the oxygen-containing functional groups on the coal surface reduce to a certain extent. This is consistent with the simulation results of the adsorption behavior of the collector on the coal surface.

## Figures and Tables

**Table 1:** Conventional analysis of the Bulianta coal sample ^1^.

Proximate Analysis (ω%)			Density (g/cm^3^)	Ultimate Analysis (ω_daf_%)				
M_ad_	A_ad_	V_daf_		C	H	O ^2^	N	S
5.84	10.53	31.26	1.39	74.33	5.23	18.97	1.03	0.44

^1^ M_ad_ is moisture mass fraction of samples on air-dried basis; A_ad_ is ash mass fraction of samples on a dry basis; V_daf_ is volatile matter mass fraction of samples on a dry and ash-free basis; and ω_daf_/%, weight percentage of various elements on a dry and ash-free basis. ^2^ By difference.

**Table 2:** The ratio of the mumber of atoms.

Atomic Ratio	H/C	O/C	N/C
**Bulianta**	0.84	0.19	0.01

**Table 3:** Structural parameters derived from ^13^C-NMR for the Bulianta coal sample ^1^.

Sample	$f_{a}$	$f_{a}^{c}$	$f_{a}^{\prime }$	$f_{a}^{H}$	$f_{a}^{N}$	$f_{a}^{P}$	$f_{a}^{S}$	$f_{a}^{B}$	$f_{al}$	$f_{al}^{*}$	$f_{al}^{H}$	$f_{al}^{O}$
**Bulianta**	62.24	1.79	60.46	33.74	26.72	7.54	6.48	12.69	37.76	6.90	27.58	3.28

^1^ Parameters: $f_{a}$, total sp2 hybridized carbons; $f_{a}^{c}$, carbonyl or carboxyl group carbons; $f_{a}^{\prime }$, aromatic carbons; $f_{a}^{H}$, protonated aromatic carbons; $f_{a}^{N}$, nonprotonated aromatic carbons; $f_{a}^{P}$, aromatic carbons bonded to hydroxyl or ether oxygen; $f_{a}^{S}$, alkylated aromatic carbons; $f_{a}^{B}$, aromatic bridgehead carbons; $f_{al}$, total sp3 carbons; $f_{al}^{*}$, methyl carbons; $f_{al}^{H}$, CH or CH2; $f_{al}^{O}$, aliphatic carbons bonded to oxygen.

**Table 4:** The type of aromatic structure in Bulianta coal.

Aromatic Unit Type	Number	Aromatic Unit Type	Number
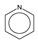	1	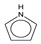	1
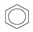	2	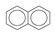	1
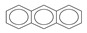	2	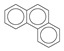	2

**Table 5:** XPS O 1s and N 1s data of the Bulianta coal sample.

Elemental Peak	Functionality	Binding Energy (eV)	Molar Content (%)
O 1s	C=O	531.2	21.8
	-OH	532.0	32.6
	C-O-C	532.7	30.5
	COO-	533.4	15.1
N 1s	pyridinic nitrogen	398.8	33.8
	pyrrolic nitrogen	400.2	43.3
	quaternary nitrogen	401.4	22.9

**Table 6:** Element composition and density of the Bulianta coal models.

Formula	Element Content	Molecular Weight
C_143_H_120_N_2_O_29_	C, 73.71%; H, 5.15%; N, 1.20%; and O, 19.93%	2328

**Table 7:** The density distribution range of water, collector, and Bulianta coal molecules along Z axis (Å).

System	Coal	Collectors	Water
water/coal	0–66.89	-	43.25–93.06
water/methyl laurate/coal	0–66.91	44.49–67.85	51.76–97.98
water/dodecanol/coal	0–66.93	44.58–69.48	44.87–97.15

**Table 8:** The self-diffusion coefficient (D) of water in the water/coal and water/collector/coal system.

System	D (10^−9^ m^2^/s)
water/coal	4.76
water/methyl laurate/coal	7.03
water/dodecanol/coal	5.42

**Table 9:** Relative contents of carbon forms on the coal surface before and after the adsorption of collectors.

E/eV	Carbon Form	Content/%		
		Raw Coal	Dodecanol	Methyl Laurate
284.8	C-C, C-H	68.9	73.93	76.85
286.3	C-O	19.8	16.66	15.49
287.5	C=O	6.40	6.48	5.08
289.1	COO-	4.90	2.53	2.58
